# Differences Between Posttraumatic Growth and Resiliency: Their Distinctive Relationships With Empathy and Emotion Recognition Ability

**DOI:** 10.3389/fpsyg.2022.825161

**Published:** 2022-03-28

**Authors:** Taylor Elam, Kanako Taku

**Affiliations:** Department of Psychology, Oakland University, Rochester, MI, United States

**Keywords:** posttraumatic growth (PTG), resilience, trauma, emotion recognition, facial expressions

## Abstract

Posttraumatic growth (PTG) and resiliency have been observed among people who experienced life crises. Given that the direct relationships between PTG and resiliency have been equivocal, it is important to know how they are different in conjunction with cognitive ability. The purpose of this study is to examine how perceived PTG and resiliency would be, respectively, associated with empathy and emotion recognition ability. A total of 420 college students participated in an online survey requiring them to identify emotions based on photographs of facial expressions, report their traumatic experiences, and respond to the PTG Inventory, Brief Resilience Scale, and Questionnaire of Emotional Empathy. The results suggest that perceived PTG was not associated with empathy but significantly predicted increased emotion recognition, whereas resiliency showed a negative relationship with empathy but no significant relationship with emotion recognition. These findings demonstrate that self-perceived PTG may be associated with cognitive ability, which could be due to one’s growth within relationships and social interactions. Even though growing after trauma may promote resilient characteristics, the current results indicate that PTG and resiliency may foster different outcomes. Since empathy and emotion recognition are affected by other contextual factors, future studies should assess how empathy and the type of errors in emotion recognition may be associated with situational factors that are beyond personal factors such as post-traumatic life experiences or personality.

## Introduction

Many individuals may face a traumatic or highly stressful event at some point in their lives. That could mean experiencing a natural disaster, the loss of a loved one, an accident or injury, or conflicts within their family and relationships. Facing adversity can have the capacity to shatter and challenge one’s core beliefs. This process can lead an individual to transform in a way that positively impacts their quality of life and helps them to realize how they have grown as a person (Tedeschi and Calhoun, 2004; Joseph and Butler, 2010; Joseph et al., 2012), known as posttraumatic growth or PTG. However, facing adversity does not always shake beliefs or make people struggle, but can rather allow them to simply bounce back, known as resiliency (Yao and Hsieh, 2019).

Posttraumatic growth explains the positive psychological changes as a result of a struggle with a major life crisis or traumatic event (Calhoun and Tedeschi, 2014; Tedeschi et al., 2018). This process may be reflected through an individual gaining a greater appreciation for life, relating to others more, making a spiritual or existential change, having an increased sense of personal strength, or realizing new possibilities in life (Tedeschi and Calhoun, 2004). After experiencing adversity, individuals may have the capacity to learn from the event and reshape the way that they perceive themselves, their lives, and their world. For example, after someone has dealt with the loss of a loved one, they may realize that their relationships with others are very important, and therefore, build stronger bonds with their family and friends. Doing so ultimately may increase their level of social support, allowing for safety and security if uncertain times were to present themselves again.

On the contrary, if an individual is able to recover from a traumatic experience by exhibiting certain characteristics (e.g., flexibility and optimism) along with using various resources that are available for them (e.g., adaptive coping strategies and social support), they could be described as being resilient. Resilience explains the likelihood that an individual can overcome highly stressful events, remaining psychologically healthy despite undergoing hardships (Rutter, 2007). Not to be misunderstood with PTG, which involves severe psychological struggle due to the challenged core beliefs following a trauma – resilience is typically understood to be the way that an individual “bounces back.” For example, someone who has experienced a stressful financial pitfall may be able to use personal resources and mechanisms (e.g., coping skills, emotion regulation, hope, optimism) to help them push through the difficult time in order to recover to the level of financial stability they were able to maintain before the decline – regaining normality in their life (Duan et al., 2015). This means that they did not allow their finances to continue to plunder and negatively impact their life, but they also did not need to overwork themselves or overthink their beliefs enough to cause them to reevaluate several aspects of their life in order to change them in a profound or transformational way. In short, resilience focuses on adapting and adjusting to adversity with or without struggling, whereas, PTG focuses on transformative changes resulting from psychological struggle caused by shattered beliefs or worldview.

Due to these conceptual differences between PTG and resiliency, the relationship between them in literature is inconsistent. At least two studies have found that there is a negative relationship between PTG and resiliency (Levine et al., 2009; Zerach et al., 2013). This could possibly be due to highly resilient people being less influenced by a trauma experience, withholding their need for growth. Other studies found that there is a positive relationship between PTG and resiliency, suggesting that the more likely someone is to experience growth after trauma, the more likely they are to exhibit characteristics of resilience as well (Bensimon, 2012; Yu et al., 2014; Duan et al., 2015). Literature has identified a curvilinear relationship between PTG and resilience which may suggest there being a possibility of a certain threshold, or “tipping point” associated with the two constructs, where the more resilient an individual is, the more likely they are to exhibit growth following adversity or vice versa, up to a certain point in which either the individual could become too resilient to experience growth or be influenced by traumatic events (Li et al., 2015; Kaye-Tzadok and Davidson-Arad, 2016). And yet, studies have also found that there is no linear relationship between PTG and resiliency (DeViva et al., 2016; Vieselmeyer et al., 2017). Given that the direct relationships between PTG and resiliency are equivocal, it is important to further investigate the distinct characteristics of each concept.

In order to reveal potentially distinct characteristics of PTG and resiliency, the current study focuses on empathy and emotion recognition ability because previous research has found that empathy is positively associated with PTG (Tedeschi and Calhoun, 2004), but little study was done for resiliency. Emotional or affective empathy is the tendency to feel the emotions of other people while keeping an other-focused and compassionate perspective. It is the ability to understand the emotions of another person that is an automatic, and often unconscious, reaction commonly understood to be the meaning behind the phrase of placing oneself in another person’s shoes (Mehrabian and Epstein, 1972). For example, a highly emotionally empathetic individual may cry during a movie where the main character’s family member has passed away. This empathetic person is able to understand the emotions of that character so well, that they exhibit or feel those emotions within themselves. It does not solely pertain to feeling sorry or having pity for someone, but it is displayed by gaining a sense of connection for what someone else may be going through or is currently feeling. A highly empathetic person is then able to use a combination of sympathy and compassion to console and approach others in a meaningful and positive way (Batson and Shaw, 1991; Pavey et al., 2012).

Findings suggest that the more growth an individual has experienced following trauma, especially growth involving others, such as within relationships, social support, and being more compassionate or more connected to others, the more likely they are to be empathetic towards them (Tedeschi and Calhoun, 2004; Cofini et al., 2014). Therefore, having experienced adversity that provoked PTG may allow an individual to be better at feeling and understanding those similar emotions in other people (Swickert et al., 2012). On the other hand, at least one study (Morice-Ramat et al., 2018) indicates that certain levels of empathy may promote more resilient characteristics, but the relationships between empathy and resiliency need to be further studied, because being able to bounce back following a trauma involves intra-personal characteristics; thus, unlike PTG, resiliency is conceptually more distant from empathy that involves inter-personal characteristics.

Emotions are essentially what prepare us to deal with or react to important events and situations without having to think deeply about them (Ekman, 1972). Not only do people feel emotions and have the capacity to understand the emotions that someone else may be feeling, but people physically express emotions as well. There are seven basic and universal emotions: anger, fear, disgust, sadness, happiness, surprise, and contempt (Ekman, 1970, 1972; Ekman and Keltner, 1997). These emotions are automatically expressed through our facial muscles when we experience them, known as facial expressions (Ekman et al., 1971). Research has found that highly empathetic people are better able to accurately identify facial expressions in others (Carr and Lutjemeier, 2005; Besel and Yuille, 2010). Empathy can be linked to mirror neurons that demonstrate neurological processes that coincide with someone’s level of empathy (Debes, 2017). This suggests that having an increased level of understanding for other individuals’ current emotional well-being is what aids in being able to read others’ emotions. The most current PTG theoretical model has identified that self-recognized PTG can be associated with outcomes that span beyond well-being, including expanded coping repertories, increased compassion, and improved wisdom – all of which aid in the development, maintenance, and improvement of interpersonal relationships (Tedeschi et al., 2018). Therefore, since empathy and similar concepts are related to PTG, accurately identifying emotion expressions could be associated with PTG as well.

On the other hand, since being highly resilient is not directly and conceptually linked to empathy, the relationship between resiliency and emotion recognition ability is also unknown. In addition to the curvilinear relationship some studies have found between PTG and resilience, resilience researchers also suggested early on that there was a need to explore whether there is a capacity or threshold in which an individual can reach that “caps” their ability to continue to adapt, adjust, or be influenced by change over their lifespan of consistently withstanding adversity (Staudinger et al., 1993; Werner, 2005). Therefore, it may be important to explore this phenomenon in connection to social perspectives, relations, and interactions. It’s possible that one’s interpersonal development, in the contexts of emotional empathy and emotion recognition, may be affected over time due to a constant resistance or recovery to hardships. Overall, the ability to accurately read the emotions of others through their body language and facial expressions is a vital skill to have in daily life. Identifying the feelings of others allows an individual to determine their actions and behaviors toward them, providing that individual with the necessary information to respond accordingly.

Revealing the relationships between PTG and emotion recognition ability is also expected to make a theoretical contribution, because PTG reports are retrospective, requiring an individual to reflect on how they were before the traumatic event which, in turn, creates discrepancies between self-reported PTG and actual growth and/or cognitive improvement (Frazier et al., 2009). It is possible that people may amplify when they estimate how they changed by having a distorted view of their growth following the trauma, or simply not know just how much of an improvement they actually made (Taylor et al., 2000). Therefore, there has been debate on whether perceived growth is an illusory concept that is susceptible to deception (Maercker and Zoellner, 2004). It is important to examine how perceived PTG is related to cognitive ability in order to establish a concrete understanding of PTG’s benefits in someone’s life. Even though PTG is conceptually linked to increased empathy levels, and empathy shares a positive relationship with emotion recognition ability (ERA), current literature has not directly examined the relationship between PTG and ERA. Experiencing growth after adverse experiences could improve cognitive processing due to the individual’s increased participation in social settings (Stephens et al., 2013) and cognitive/emotional processing that they are engaged with. Therefore, the current study aimed to investigate the relationships between perceived PTG, resilience, empathy, and ERA. Given that this is the first study that investigates the associations among all these variables, no specific hypotheses were generated. However, due to the equivocal association between PTG and resilience, we expected that the size and direction of the relationships between PTG and empathy/ERA would be different than the relationships between resilience and empathy/ERA.

## Materials and Methods

### Participants and Procedure

The sample consisted of 420 undergraduate students at a midwestern university in the US who had a mean age of 21.04 years (*SD* = 5.15). Approximately 65% of participants identified as White, 12% as African American, 10% being of Middle Eastern Heritage, 7% as Asian, and 5% identified as other. Additionally, about 80% of the sample were female and 19% were male. Two of the participants (less than 1%) did not provide their sex.

Participants were recruited through a university’s subject pool and received class credit upon completion. They were first asked to provide demographic information and to identify emotions based on photographs of facial expressions. They then completed a questionnaire regarding empathy, which was followed by identifying their trauma experiences and PTG. Lastly, they completed a questionnaire measuring resilience. The study was approved by an internal review board (IRB-FY2020-16). Data were analyzed using SPSS 26.

### Measures

#### Traumatic Events

Participants indicated which out of 13 traumatic events (e.g., “natural disaster,” “accident or injury,” “death of someone close to you”) they had experienced in the last five years, a measure that has been used in previous research (Taku, 2011). Following the trauma checklist, they identified which event impacted them the most (Taku, 2013).

#### Posttraumatic Growth

The PTG Inventory-Short Form (PTGI-SF; Cann et al., 2010; α = 0.91) was used to measure the participants’ level of perceived PTG as a result of the traumatic event that most impacted them (e.g., “I changed my priorities about what is important in life”). For 10 items, the participants were asked to indicate the degree to which each change had occurred for them on a 6-point Likert scale ranging from 0, “not at all,” to 5, “very great degree.” Participants that did not identify a trauma event (*n* = 10) were excluded when analyzing total perceived PTG scores.

#### Resilience

The Brief Resilience Scale (BRS; Smith et al., 2008; α = 0.86) with 6 items (e.g., “I tend to bounce back quickly after hard times”) was rated on a 5-point Likert scale ranging from 1, “strongly disagree,” to 5, “strongly agree.”

#### Empathy

The Questionnaire Measure of Emotional Empathy (QMEE; Mehrabian and Epstein, 1972; α = 0.87) with 33 items (e.g., “it makes me sad to see a lonely stranger in a group”) was rated on a 9-point Likert scale ranging from 1, “very strong disagreement,” to 9, “very strong agreement.”

#### Emotion Recognition

The Standard Expressor Version of the Japanese and Caucasian Facial Expressions of Emotions (JACFEE; Matsumoto and Ekman, 1988; Ekman and Matsumoto, 1993) was used to measure an individual’s ability of identifying the seven universal emotions: anger, disgust, contempt, fear, happiness, sadness, and surprise. The original set consists of a total of 130 photographed expressions from nine expressers (i.e., five Caucasian males, three Caucasian females, and one Japanese male). However, to diversify the measure as much as possible, as well as account for burnout and online efficiency, only a total of 24 photographs were used; a set of 8 facial expressions (i.e., anger, contempt, disgust, fear, happiness, sadness, surprise, and neutral) from each of 3 expressers (i.e., one Japanese male, one Caucasian female, and one Caucasian male). The 24 photographs were presented in a randomized order where the participants were asked to answer, “what emotion is this person expressing?” The amount of expressions identified correctly out of the 24 emotion expressions was used for a total ERA score. Only participants that answered all 24 items were included when analyzing ERA.

## Results

### Posttraumatic Growth, Resilience, and Empathy

As shown in Table 1, a weak positive relationship was found between PTG and resilience, *r* = 0.19, *p* < 0.01. PTG and empathy were not correlated with one another (*r* = 0.09, *p* = 0.08), but resilience and empathy were found to be negatively correlated with one another, *r* = −0.34, *p* < 01.

**TABLE 1:** Correlations of PTG, resilience, and empathy.

	1.	2.	3.	Score Range	M (SD)	α
(1). PTG	-			0 – 50	30.98 (11.34)	0.91
(2). Resilience	0.19***	-		6 – 30	18.46 (4.87)	0.86
(3). Empathy	0.09	−0.34***	-	33 – 297	200.88 (26.60)	0.87

***p < .001, PTG = Posttraumatic growth.

### PTG, Resilience, Empathy and Emotion Recognition Ability

Unlike self-perceived scales (i.e., PTG, resilience, and empathy), ERA reflects cognitive abilities through the expression and identification of universal emotions, and therefore, our participants were able to recognize more than half of the emotions accurately, leading to a non-normal distribution. Due to that, the mean score of ERA, 19, was used as a cutoff to create two groups: an ERA-low group (*n* = 144) that identified less than 19 emotions out of the 24 correctly, and an ERA-high group (*n* = 245) that identified 19 or more emotions correctly. A logistic regression model was created to test the likelihood that PTG and resilience would predict ERA group differences. As displayed in Table 2, the model as a whole was statistically significant: *X*^2^(2, *N* = 372) = 7.38, *p* = 0.03. This indicated that approximately 2% of the variance of ERA can be explained by PTG and resilience. However, only PTG showed to significantly predict (*p* = 0.02) differences between the ERA-low group and the ERA-high group. Whereas, resilience showed to make no significant contribution in predicting ERA group differences (*p* = 0.10).

**TABLE 2:** Logistic regression analysis for PTG and resilience predicting ERA groups.

		*B*(SE)	*p*	95% CI
Model 1 (*N* = 372)	PTG	0.02(.01)*	0.02	[1.01, 1.04]
	Resilience	−0.04(.02)	0.10	[0.92, 1.01]

Model 1: R^2^ = 0.02 (Cox and Snell), 0.03 (Nagelkerke). Model X^2^(2) = 7.38, p = 0.03. *p < .05. PTG = Posttraumatic growth. ERA = Emotion Recognition Ability. ERA Groups: Low = Participants who answered less than 19 emotions correctly out of 24, High = Participants who answered 19 or more emotions correctly out of 24.

## Discussion

The current study was designed to examine the relationships between PTG, resiliency, empathy, and ERA. Specifically, we investigated the ways in which PTG and resiliency may be different by analyzing their potential distinctive relationships with empathy and one’s accuracy in identifying facial expressions. Overall, PTG and resiliency were found to have a significant but weak positive relationship, and their respective relationships with both empathy and emotion recognition were different.

First, PTG and empathy were found to be uncorrelated. This suggests that positive changes an individual perceives as a result of a trauma, such as appreciating life, having more compassion, and being able to do better things in life, and their ability to understand how others feel were independent from each other. This may be because PTG includes multiple domains, ranging from content that is not directly related to empathy, such as finding new opportunities that would not have been available without the specific triggering event, to content that should be related to empathy, such as being more compassionate for others, and they might cancel each other out.

On the other hand, empathy and resilience showed a negative relationship. This could explain that the more resilient someone is, the less empathetic they are or vice versa. Since one study has suggested that empathy could be a predisposition of resilience (Morice-Ramat et al., 2018), the current findings may suggest that the more resilient people become, the less likely they may be able to relate and share emotional experiences with others – or perhaps, that the more empathic they are, the less resilient they are. It is possible that there are other factors, such as self-sufficiency, autonomy, self-confidence or toughness, that may cause these constructs to be inversely related with one another. The heightened ability to continuously overcome obstacles may cause highly resilient people to develop and remain at an emotional equilibrium, not being heavily influenced by certain situations or susceptible to others’ emotional states, and therefore, making them less sensitive to others who may be strongly influenced by their daily circumstances that causes them to both feel and express a wide range of emotions. These results of inconsistent relationships with empathy indicate PTG and resilience differ.

Second, the current study suggested that PTG, but not resiliency, predicted emotion recognition ability. More specifically, PTG significantly predicted ERA group differences, where higher perceived PTG levels were more likely to be associated with belonging to the ERA-high group, explaining that higher growth is more likely to lead to increased emotion recognition. This suggests that perceived PTG may not be entirely illusory, since it was associated with the cognitive abilities of identifying emotions on pictures, which is unrelated to each person’s life narratives. On the other hand, resilience did not significantly predict ERA group differences, suggesting that being resilient and cognitive abilities in reading others’ emotions are independent from each other. Even though someone highly resilient may show a lower level of empathy, that does not necessarily mean that their ability to identify emotions in others is also low, since the results showed no significance. It is important to note, however, due to the non-normal distribution of ERA scores, participants were assigned into the two ERA groups (low-high) using the cutoff of 19 for this study, which means some of the participants in an ERA-“low” group were still able to identify 75% of the emotions correctly (e.g., 18 out of the 24 pictures). Similarly, some of the participants in an ERA-“high” group were only able to identify 83% of the emotions correctly (E.g., 20 out of 24 pictures); thus, they showed a few errors as well.

## Implications, Limitations and Future Directions

Posttraumatic growth and resiliency are both processes that one may experience following a potential traumatic event that share similar characteristics, but are also very distinct from one another. Both growing and bouncing back after adversity are positive constructs but it is important to understand the potential differences. This study lends insight into the ways in which PTG was related to ERA but not empathy, but when it comes to resilience, a decrease in empathy with no conclusive relationships with emotion recognition accuracy.

Empathy and ERA are important because they provide the knowledge someone needs that allows them to respond to others in the most constructive way. For example, an empathetic person who is also fairly good at recognizing emotions is able to notice that their friend is sad based on their facial expressions and then empathize with them because they know what sadness feels like. Since they have the knowledge to accurately identify that their friend is sad and use their previous life experiences to understand their sadness, they are able to recall what it is they may have needed from someone when they were in the same position. They may recall that in their own time of sadness, they desired a hug from their loved one or wanted to talk about what caused them to feel that way. Due to that understanding, they are able to react to their friend in a similar way. This may then provide comfort and support to their friend, increasing the quality of their relationship which would lead to a stronger bond between them. Being both empathetic and high in person perception allows an individual to notice behaviors and resonate with them, further allowing someone to respond in an appropriate manner. Strong interpersonal skills are necessary for effectively communicating, connecting, and collaborating with others, prospering in professional matters, as well as developing and maintaining a safe and secure social support system – which can all result in a good quality of life and overall well-being.

The more independent, intrapersonal nature of resilience may be the biggest aspect in which it differs from PTG. Resilience causes one to call upon personal skills and characteristics to recover from tragedy, however, PTG causes one to do that but in addition to changing the way in which they relate, interact, and express themselves with others, recently demonstrated in a cross-cultural study that showed having positive experiences when disclosing a trauma to others is the only significant predictor for PTG across 10 countries (Taku et al., 2021). PTG may be more realized when the experience was shared with at least one person who can be there for them, whereas resiliency may be more recognized without the presence of others. Programs that stress the importance of being resilient and programs that stress the importance of transformational growth may be able to complement each other well. Focusing on implementing practices that involve becoming more empathic toward others may benefit individuals who are highly resilient. The applications of PTG can now highlight not only leading to feeling stronger, appreciating life more, becoming spiritually connected, realizing new possibilities in life, and developing stronger bonds with others, but may include cognitive abilities in judging others’ emotions. This study provides evidence showing that perceived PTG may not be entirely illusory, and may portray quantifiable objective positive changes in terms of emotion recognition accuracy.

Even though this study provides insight into the relationships between PTG, resiliency, empathy, and ERA, there are limitations. The lack of diversity within the sample demographics such as age, race, and gender, makes it difficult to generalize these findings to various other populations. Due to the lack of timing how long it took the participants to identify the facial expressions, the participants had more time to ruminate, which could have contributed to the overall high accuracy rate. Lastly, the self-report online nature of the study makes the research susceptible to inaccuracy, however, the sample size is substantial enough to buffer against most inaccuracies.

Despite these limitations, this study has fostered further exploration into the differences between PTG and resiliency. Specifically, we identified that the factors that can distinguish PTG and resiliency may be within interpersonal constructs such as empathy and ERA. Future research should explore what other factors can help explain the differences as well as overlaps between PTG and resiliency. Furthermore, it is important to investigate what types of growth may lead to cognitive improvement over others along with which emotions (e.g., anger, sadness) are easier to identify over others among people who experienced PTG as opposed to people who are highly resilient. Expanding this study to a wider audience of various different backgrounds would make the findings more applicable and generalizable to more populations. Research should also explore whether the amount/type of trauma events experienced affects an individual’s PTG, resilience, empathy, and emotion recognition ability. Replicating this study with the addition of a cognitive empathy measure, a more diverse expressor measure, and a timing feature for identifying the facial expressions, would further provide insight into the relationships between PTG, resiliency, empathy, and emotion recognition.

In summary, the current study identified how characteristics of PTG and resiliency may differ from one another through interpersonal contexts such as with empathy and identifying facial expressions. These findings suggest that it may be important to acknowledge the potential differences between PTG and resiliency and continue to pinpoint the ways in which PTG and resiliency can be better understood in order to properly approach, teach, and implement these constructs.

## Data Availability Statement

The original contributions presented in the study are included in the article/supplementary material, further inquiries can be directed to the corresponding author/s.

## Ethics Statement

The studies involving human participants were reviewed and approved by Oakland University Institutional Review Board. The patients/participants provided their written informed consent to participate in this study.

## Author Contributions

TE and KT: conception and design of the study and acquisition of data and drafting the manuscript and tables. TE: analysis of the data. Both authors have reviewed and edited the manuscript.

## Conflict of Interest

The authors declare that the research was conducted in the absence of any commercial or financial relationships that could be construed as a potential conflict of interest.

## Publisher’s Note

All claims expressed in this article are solely those of the authors and do not necessarily represent those of their affiliated organizations, or those of the publisher, the editors and the reviewers. Any product that may be evaluated in this article, or claim that may be made by its manufacturer, is not guaranteed or endorsed by the publisher.
